# COVID-19’s Impact on International Trade

**DOI:** 10.3390/e24030327

**Published:** 2022-02-24

**Authors:** Célestin Coquidé, José Lages, Leonardo Ermann, Dima L. Shepelyansky

**Affiliations:** 1 Institut UTINAM, OSU THETA, Université Bourgogne Franche-Comté, CNRS, 25000 Besançon, France; celestin.coquide@utinam.cnrs.fr; 2 Departamento de Física Teórica, GIyA, Comisión Nacional de Energía Atómica, Av. del Libertador 8250, Buenos Aires 1429, Argentina; ermann@tandar.cnea.gov.ar; 3 Consejo Nacional de Investigaciones Científicas y Técnicas (CONICET), C1425FQB, Buenos Aires 1650, Argentina; 4 Laboratoire de Physique Théorique, IRSAMC, Université de Toulouse, CNRS, UPS, 31062 Toulouse, France; dima@irsamc.ups-tlse.fr

**Keywords:** COVID-19, international trade, complex networks, PageRank, Google matrix

## Abstract

We analyze how the COVID-19 pandemic affected the trade of products between countries. With this aim, using the United Nations Comtrade database, we perform a Google matrix analysis of the multiproduct World Trade Network (WTN) for the years 2018–2020, comprising the emergence of the COVID-19 as a global pandemic. The applied algorithms—PageRank, CheiRank and the reduced Google matrix—take into account the multiplicity of the WTN links, providing new insights into international trade compared to the usual import–export analysis. These complex networks analysis algorithms establish new rankings and trade balances of countries and products considering all countries on equal grounds, independent of their wealth, and every product on the basis of its relative exchanged volumes. In comparison with the pre-COVID-19 period, significant changes in these metrics occurred for the year 2020, highlighting a major rewiring of the international trade flows induced by the COVID-19 pandemic crisis. We define a new PageRank–CheiRank product trade balance, either export or import-oriented, which is significantly perturbed by the pandemic.

## 1. Introduction

The COVID-19 pandemic produced an enormous impact on human society with multiple health, social and economic effects [1]. The impact on international trade has been enormous. Thus, the World Trade Organization (WTO) states that “the COVID-19 pandemic represents an unprecedented disruption to the global economy and world trade, as production and consumption are scaled back across the globe” [2]. Several reports of the WTO and the United Nations (UN) highlight the effects of COVID-19 for world trade [3,4,5]. A need for an effective public health response to the collapse of the global trade is elucidated in [6].

Recent papers were devoted to the study of the effects of COVID-19 on international trade. Using the concept of supply chain resilience, in [7], the authors defined the interconnectedness between country-level factors that permitted countries to be resilient during the pandemic period and more especially during the first wave. Their analysis of countries’ resilience is based on a Fuzzy-set Qualitative Comparative Analysis (fsQCA), permitting them to list factors giving countries the best chance to survive and recover. Multiple factors have been chosen, such as the trade resilience (based on 2019–2020 monthly WTO data), logistics performance (index measured by the world bank), healthcare preparedness (global heath security) and others. In [8], the direct impact of the COVID-19 pandemic on global trade has been analyzed through the 2019 and 2020 export reports obtained from the IHS Markit company Global Trade Atlas. Using a gravity model that takes into account the number of deaths/cases and the closure of workplaces, it has been shown [8] that exporters were negatively impacted since transportation was limited due to the lockdowns and the closure of workplaces. Although importers have also suffered from a negative impact, the latter decreased rapidly after the first epidemic wave in the case of first-necessity commodities such as food and medical products. An analogous study [9] reports that e-commerce mitigated the negative impact for importing countries. An interesting debate emerged from early studies [10,11,12] about the simulations of lockdown scenarios and their impacts on global trade.

The WTO and the UN reports [2,3,4,5] depict the global change of world trade induced by COVID-19. However, it is also important to analyze how the trade relations between the countries are structurally affected by the pandemic. With this aim, we perform a Google matrix analysis of the World Trade Network (WTN) constructed from the UN Comtrade database [13] for the years 2018, 2019 and 2020. The WTN concerns $N_{p}=10$ types of commercial products (see Table 1) traded between $N_{c}=194$ world countries. After the pioneering work of Brin and Page [14], the invented PageRank algorithm, based implicitly on the Google matrix construction, has found broad applications for the World Wide Web (WWW) search engines [14,15]. In the last decades, human society has created a variety of complex networks whose main properties are described, for example, in [16]. The applications of Google matrix algorithms to various directed networks include university web sites and Wikipedia networks [17,18], Linux Kernel networks [19,20], networks of protein–protein interactions [21,22,23] and many others.

The reader can find an overview of the typical properties of the complex networks in [16]. The generic properties and the mathematical aspects of the Google matrix associated with directed complex networks are described in [14,15,16].

The first applications of Google matrix methods to the UN Comtrade-based WTN have been presented in [24,25]. While only the PageRank algorithm was used in [24], it was shown in [25] that it is equally important to use the CheiRank algorithm, which describes the outgoing trade flows. Indeed, while the PageRank describes the ingoing trade flows—i.e., the import flows—the CheiRank, explained in detail in [17,19], describes the export flows. For the WTN study, it is clear that the combination of both the PageRank and the CheiRank analyses is of primary importance as it extends the traditional import–export analysis by taking account of the multiplicity of cascades of trade exchanges. The further development of Google matrix applications to the WTN allowed researchers to perform the analysis of the multiproduct WTN [26] by considering the contributions of the products proportionally to their trade volumes and treating countries on equal grounds, which corresponds to the fundamental principle of the UN. These methods were also successfully applied to the networks of economic activities provided by the WTO [27]. In addition, the reduced Google matrix (REGOMAX) algorithm has been applied to the multiproduct WTN [28] and to the multisectorial trade network [29]. This algorithm has been developed and numerically tested in [30,31,32]. For a subset of interest, constituted by a moderate number $N_{r}$ of network nodes, this algorithm finds the effective interactions between these $N_{r}$ nodes taking into account all the indirect pathways passing through the global network of a much larger size $N\gg N_{r}$. For the here-studied WTN case, the size of the global network is $N=N_{c}N_{p}=194\times 10=1940$. Using the above-cited algorithms, we analyze here the impact of COVID-19 on the WTN for the years 2018–2020.

In the last couple of years, there has been a growing interest in the network analysis of the influence of COVID-19 on international trade (see e.g., [33,34,35,36]). All of these previous works, usually based on standard centrality measures such as the node degree or betweenness centralities, only consider the import flows network. However, the import flows network and export flows network, which are structurally different, are equally important; e.g., the study [33] was focused on the analysis of the PageRank (analogous to the ImportRank), while the CheiRank (analogous to the ExportRank) was not considered. The above-cited previous works consider either the global trade network constructed by summing or averaging the economic flows between each pair of countries or a specific economical sector; e.g., the cereal trade in [35]. Here, in the present work, we take into account the trade of all commodities, gathered into the 10 product groups of the Standard International Trade Classification (SITC) (see Table 1). The constructed multiproduct WTN allows us to take account of the relative economical importance of each product. In addition, many of the previous works focused their analysis on the trade between either a dozen economically leading countries or a specific set of countries; e.g., in [34] with the Association of Southeast Asian Nations (ASEAN) countries. Here, we consider the trade transactions yearly reported in the UN Comtrade database of all the commodities between 194 countries and territories. The new advantage of our approach is due to the Google matrix analysis of the WTN [18,25,26,27,28,29], which allows us to treat multiproduct commercial trade flows between world countries, taking account of ingoing and outgoing flow directions and considering all the countries on equal grounds independent of their wealth. The REGOMAX method developed in [28,30,31] allows us to highlight interdependencies between specific products and countries. For all these reasons, our global Google matrix analysis, including the REGOMAX algorithm, allows us to obtain a much deeper characterization of the impact of COVID-19 on the WTN.

We note that the methods of statistical physics and network analysis in econophysics have attracted growing interest (see, e.g., [37,38,39,40,41]), and we hope that our studies will bring new elements to such investigations. The WTN describes monetary flows of products between countries, viewed as information flows related to the entropy, which links our studies to the fundamental aspects of the Markov chains and their statistical description.

The structure of the manuscript is the following: in Section 2, we present the construction of the WTN from the UN Comtrade data and the different algorithms used for the complex network analysis. In Section 3, we present our results concerning the impact of COVID-19 on international trade, focusing on the relative ranking position of the countries on the PageRank–CheiRank plane, the global and per product trade balances and the rewiring of the WTN induced by the pandemic.

## 2. Datasets and Google Matrix Algorithms

We use the UN Comtrade data [13] for the years 2018, 2019 and 2020 (collected in September 2021) to construct the trade flows of the multiproduct WTN following [26,28], giving a detailed description of the applied methods. For each year, a money matrix, $M_{cc^{\prime }}^{p}$, gives the export flow of product *p* from country $c^{\prime }$ to country *c* (transactions are expressed in USD of the current year). The money matrix element, $M_{cc^{\prime }}^{p}$, is taken as the maximum between the reported volume of product *p* imported by the country *c* from the country $c^{\prime }$ and the reported volume of product *p* exported from the country $c^{\prime }$ to the country *c*. Although these volumes should be of the same order, this procedure allows us to reduce the effect of the absence or delay of reporting by certain countries. The data set concerns $N_{c}=194$ countries and territories and $N_{p}=10$ principal groups of products, as reported in Table 1. Thus, the total Google matrix *G* size is $N=N_{c}N_{p}=1940$ corresponding to the number of couples (product, country).

The Google matrix elements $G_{ij}$ of direct trade flows are constructed in the manner described in detail in [26,28]. The monetary values of products traded from a node *j* to the other nodes *i*, renormalized in such a way that their sum is equal to one, constitute the elements $S_{ij}$ of the matrix *S*, which represents Markov trade transitions. Each column associated with a dangling node—i.e., a node from which no trade flows emanate—is replaced by a column with all elements equal to 1/N. A personalization vector *v* encodes the relative weight of each product in the global trade volume, and all the countries are treated on equal grounds following the main UN principle. The Google matrix elements are $G_{ij}=\alpha S_{ij}+\left(1-\alpha \right)v_{i}$, where $v_{i}$ is the personalization vector component of node *i* ($v_{i}>0,\;\forall i$ and $\sum_{i=1}^{N}v_{i}=1$). Here, we use the damping factor value α=0.5. Similarly, we also construct the Google matrix $G^{*}$ from the money matrix with inverted trade directions.

The stationary probability distribution on the WTN is given by the PageRank vector *P* defined by the eigenproblem GP=P [14,15,18]. For the WTN network with inverted trade flows, described by $G^{*}$, the stationary probability distribution is given by the CheiRank vector $P^{*}$, defined as $G^{*}P^{*}=P^{*}$. The PageRank node index *K* and the CheiRank node index $K^{*}$ are obtained by sorting in descending order of the components of the PageRank vector *P* and of the CheiRank vector $P^{*}$, respectively. For the WTN, each node corresponds to a given couple $\left(p,c\right)$ of a country *c* and product *p*. Hence, the PageRank (CheiRank) component $P_{pc}$ ($P_{pc}^{*}$) measures the ability of a country *c* to import (export) a product *p*. Following [26,28], the sums over all the product types *p* give the PageRank probability $P_{c}=\sum_{p}P_{pc}$ and the CheiRank probability $P_{c}^{*}=\sum_{p}P_{pc}^{*}$ of a given country *c*. Similarly, the sums over the countries provide the PageRank and CheiRank probabilities $P_{p}=\sum_{c}P_{pc}$ and $P_{p}^{*}=\sum_{c}P_{pc}^{*}$ of a product *p*. Sorting these probabilities in descending order allows us to define the related indexes $K_{c}$, $K_{c}^{*}$, $K_{p}$ and $K_{p}^{*}$. From the import and export volumes, it is possible to define, for a couple $\left(p,c\right)$, an ImportRank probability ${\hat{P}}_{pc}=V^{-1}\sum_{c^{\prime }}M_{cc^{\prime }}^{p}$ and an ExportRank probability ${\hat{P}}_{pc}^{*}=V^{-1}\sum_{c}M_{cc^{\prime }}^{p}$, where $V=\sum_{p,c,c^{\prime }}M_{cc^{\prime }}^{p}$ is the total volume of the WTN. Analogously to the PageRank and CheiRank probabilities, it is possible to define the ImportRank and the ExportRank probabilities ${\hat{P}}_{c}$ and ${\hat{P}}_{c}^{*}$ for the country *c*, and ${\hat{P}}_{p}$, and ${\hat{P}}_{p}^{*}$ for the product *p*. Furthermore, we define $\hat{K}$ and ${\hat{K}}^{*}$ as the ImportRank and the ExportRank node index, and the other indexes ${\hat{K}}_{c}$, ${\hat{K}}_{c}^{*}$, ${\hat{K}}_{p}$ and ${\hat{K}}_{p}^{*}$. Qualitatively, the PageRank probabilities are proportional to the volumes of ingoing trade flow and the CheiRank probabilities to the volume of outgoing flow. Thus, approximately, products and countries subject to high importations (exportations) have a high PageRank (CheiRank) probability. Let us note that, in contrast to the usual import–export description—i.e., the one obtained with the ImportRank and the ExportRank, which takes only account of bilateral direct trade exchanges—the PageRank and CheiRank description takes into account the multiplicity of all the cascades of transactions encoded in the WTN.

Following [26,28], we determine the PageRank–CheiRank trade balance of a given country *c* as $B_{c}=\left(P_{c}^{*}-P_{c}\right)/\left(P_{c}^{*}+P_{c}\right)$. In a similar way, the ImportRank–ExportRank trade balance is ${\hat{B}}_{c}=\left({{\hat{P}}^{*}}_{c}-{\hat{P}}_{c}\right)/\left({{\hat{P}}^{*}}_{c}+{\hat{P}}_{c}\right)$. The sensitivity of the trade balance $B_{c}$ to the price of a given product *p* is obtained by multiplying the corresponding money matrix elements ${\left\{M_{c^{\prime }c^{\prime \prime }}^{p}\right\}}_{\forall c^{\prime },c^{\prime \prime }}$, by a factor (1+δ), where δ is an infinitesimal number. The PageRank–CheiRank trade balance sensitivity is then defined as $dB_{c}/d\delta$. It is also useful to determine the PageRank–CheiRank product *p* trade balance, defined as $B_{p}=\left(P_{p}^{*}-P_{p}\right)/\left(P_{p}^{*}+P_{p}\right)$, which is, a priori, non zero, in contrast to the ImportRank–ExportRank product *p* trade balance ${\hat{B}}_{p}=\left({\hat{P}}_{p}^{*}-{\hat{P}}_{p}\right)/\left({\hat{P}}_{p}^{*}+{\hat{P}}_{p}\right)$, which is equal to zero due to the existing symmetry of product *p* transactions ${\hat{P}}_{p}={\hat{P}}_{p}^{*}$. Finally, the product *p* trade balances of a given country *c* are defined as $B_{pc}=\left(P_{pc}^{*}-P_{pc}\right)/\left(P_{p}^{*}+P_{p}\right)$ and ${\hat{B}}_{pc}=\left({\hat{P}}_{pc}^{*}-{\hat{P}}_{pc}\right)/\left({{\hat{P}}^{*}}_{p}+{\hat{P}}_{p}\right)$.

We also use the REGOMAX algorithm [28,30,31] to obtain the reduced Google matrix $G_{R}$ associated with a selected subset of $N_{r}$ WTN nodes (with $N_{r}\ll N$). This algorithm allows us to incorporate all the direct and indirect transaction pathways connecting any couples of nodes among the $N_{r}$ nodes of interest. The reduced Google matrix $G_{R}$, obtained from the full WTN Google matrix *G*, is used to construct a reduced network summarizing the strongest trade interactions between a selection of nodes representing countries and products.

More detailed descriptions of the Google matrix based algorithms and the related numerical methods can be found in [25,26,28,30,31].

Let us note that the above-depicted algorithms have been used in [42,43] to analyze the multiproduct WTN for years before 2019, and hence, the effects of COVID-19 on the WTN have not been captured there.

## 3. Results

### 3.1. World Countries on the PageRank–CheiRank Plane

**Figure 1:**
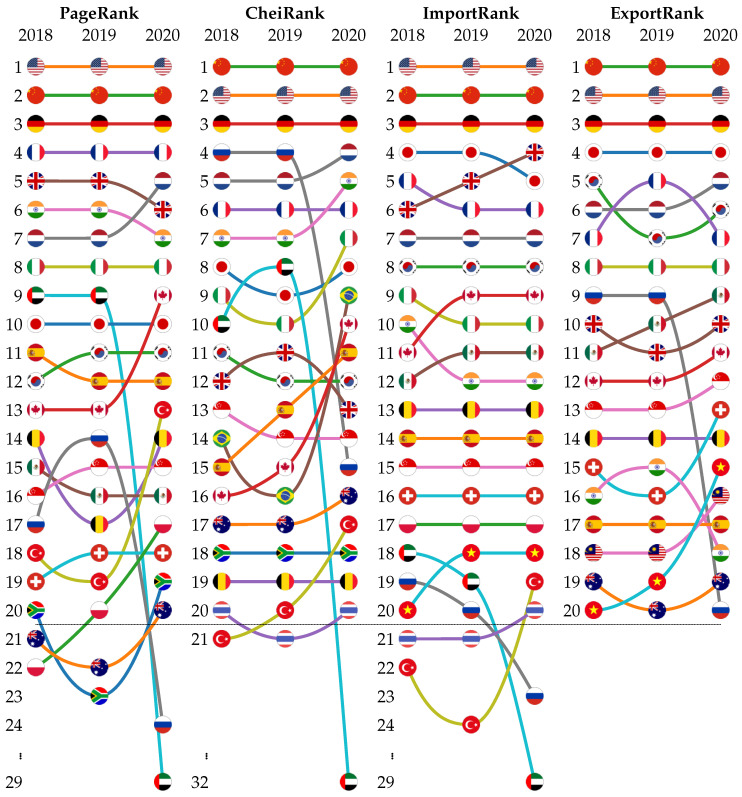
Top 20 of countries ranked according to the PageRank, CheiRank, ImportRank and ExportRank algorithms for the years 2018, 2019 and 2020. The countries are represented by their national flag.

The top 20 countries according to the PageRank, CheiRank, ImportRank and ExportRank are given in Figure 1 for the years 2018, 2019 and 2020. For each ranking, the top three countries are unchanged for the three consecutive years. The top three countries are either the USA, China and Germany for import-related rankings—i.e., ImportRank and PageRank—or China, the USA and Germany for export-related rankings—i.e., ExportRank and CheiRank. Globally, along this time period, the ImportRank and the ExportRank are quite stable for the top 20 main international trade players, suggesting that their relative total trade capacities were not significantly affected by COVID-19. Indeed, from 2018 to 2020, we observe 10 countries for which the rank did not change, while the rank of the others changed by at most ±2 positions. However, there are the following exceptions: Russia, which significantly worsened in terms of both its ImportRank ($\hat{K}=19\to 23$) and ExportRank (${\hat{K}}^{*}=9\to 20$); the United Arab Emirates (UAE), which worsened in terms of its ImportRank ($\hat{K}=18\to 29$—the UAE does not even appear in the top 20 exporters) and Vietnam, which improved its ExportRank (${\hat{K}}^{*}=20\to 15$). The relative loss of export volume for Russia and the UAE can be attributed to the significant global reduction of world production and also of air traffic induced by the COVID-19 lockdown. This automatically reduced the global requests for mineral fuels (petrol, gas…) which dominate the trade flows of Russia and the UAE. In contrast, the Vietnamese economy was one of the most resilient during the COVID-19 crisis [44].

In contrast to the import–export description, based only on the relative amount of total export and import volumes, the PageRank and CheiRank description takes account of the structure of the WTN and consequently allows us to rank countries according to their relative efficiency to import and export commodities. Roughly speaking, countries importing from (exporting to) a large variety of countries will obtain a better PageRank (CheiRank) than ImportRank (ExportRank); e.g., India, in 2018, which has the 16th export volume (10th import volume), occupies the $K^{*}=7$ (K=6) position in the CheiRank (PageRank), suggesting a more important role in the WTN structure than would be inferred by focusing only on the import–export point of view. For 2018, we can observe similar cases for the UAE ($\hat{K}=18\to K=9$, ${\hat{K}}^{*}=21\to K^{*}=10$) and for Russia (${\hat{K}}^{*}=9\to K^{*}=4$). Taking the example of Russia, the diversity of its trade relations for mineral fuels, involving both European and Asian countries, is much better captured by the PageRank–CheiRank picture, which takes account of the complexity of the WTN and the multiplicity of cascades of commercial links. Conversely, despite the fact that Japan’s import and export volumes were the fourth most important in 2018, Japan’s position dropped to K=10 in PageRank and $K^{*}=8$ in CheiRank, suggesting that the centrality of Japan in the WTN is weaker than expected, certainly due to a lack of diversity of commercial partners.

The PageRank–CheiRank picture allows us to detect a rewiring of the WTN due to the COVID-19 pandemic. Indeed, from Figure 1 (two first columns), we observe more abrupt variations from 2019 to 2020 (up to +6 and −20 positions for the PageRank of Turkey and UAE, respectively; up to +7 and −22 positions for the CheiRank of Brazil and UAE, respectively) than from 2018 to 2019 (up to +3 and −3 positions for the PageRank of Russia and Belgium, respectively; up to +2 and −2 positions for the CheiRank of, e.g., Spain and Brazil, respectively). Mimicking the loss of import and export volumes experienced by Russia and the UAE from 2018 to 2020, Russia’s CheiRank (PageRank) drops by 11 (7) positions and the UAE’s CheiRank (PageRank) drops by 22 (20) positions. Excepting these two cases explained by extreme variations of trade volumes, we also observe for some other countries quite large variations from 2018 to 2020; e.g., Brazil gains 5 positions in CheiRank, climbing up to $K^{*}=9$, whereas its volume of export stays stable around the 24th position (${\hat{K}}^{*}=24$ in 2018 and 2019, ${\hat{K}}^{*}=23$). A similar gain is observed for Canada ($K^{*}=16\to 10$), but with a higher export volume (${\hat{K}}^{*}=12$ in 2018 and 2019, ${\hat{K}}^{*}=11$). India also gained +2 CheiRank positions, which can be attributed to its broad trade connections with Asia, Europe and America and an enhanced export of variety of products—e.g., pharmaceutics—during the COVID-19 pandemic. Among European countries, we note that the Netherlands, Italy and Spain improved their CheiRank positions from 2018 to 2020.

**Figure 2:**
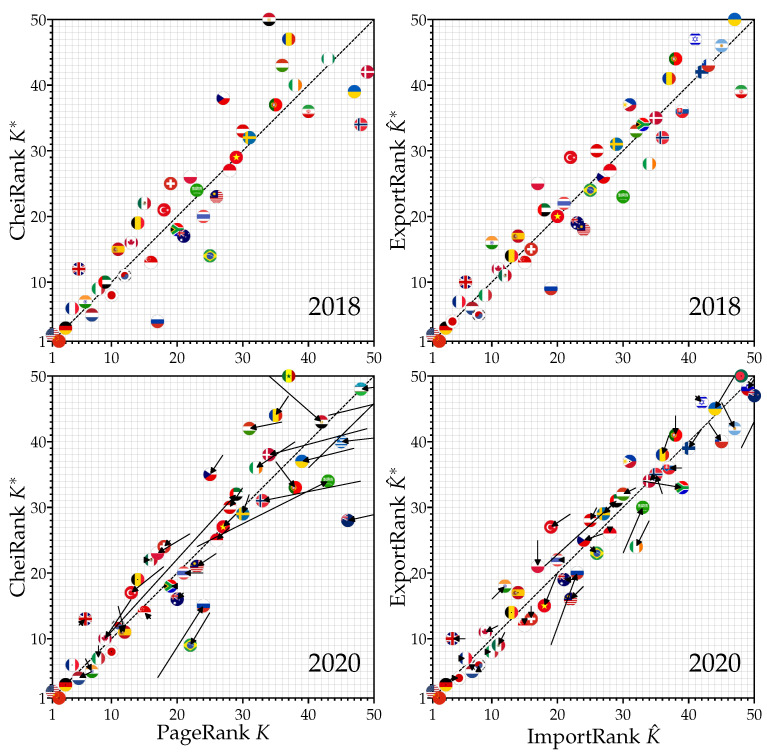
Distributions of countries on the PageRank *K*–CheiRank $K^{*}$ plane (**left** panels) and on the ImportRank $\hat{K}$–ExportRank ${\hat{K}}^{*}$ (**right** panels). The distributions are shown for the years 2018 (**top** panels) and 2020 (**bottom** panels). On the bottom panels, each arrow indicates the change between the two years: the arrow tail [head] is located on the point $\left(K,K^{*}\right)$ for 2018 [2020]. The countries are represented by their corresponding national flag.

**Figure A1:**
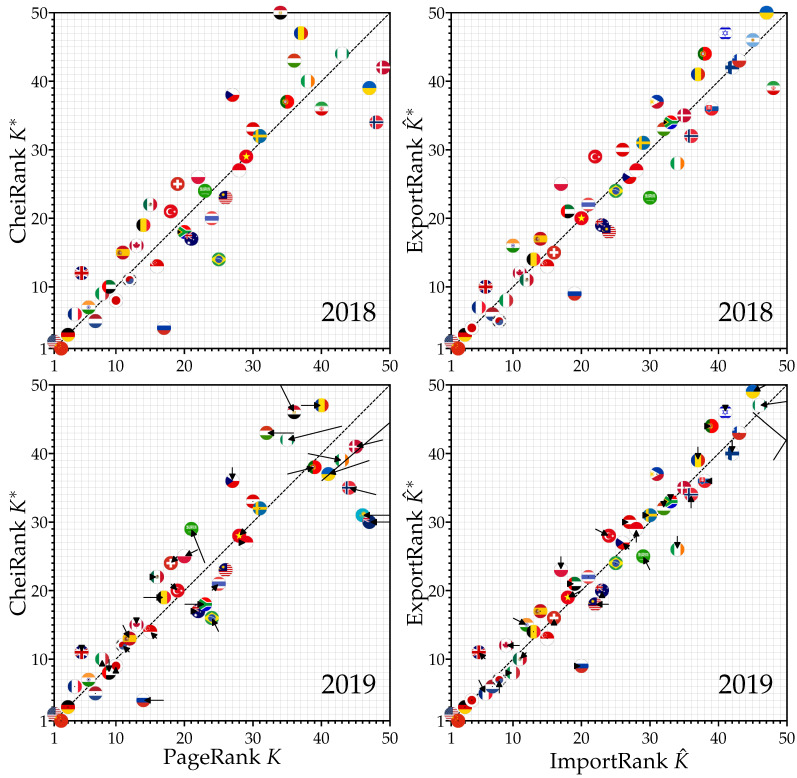
Distributions of countries on the PageRank *K*–CheiRank $K^{*}$ plane (**left** panels) and on the ImportRank $\hat{K}$–ExportRank ${\hat{K}}^{*}$ (**right** panels). The distributions are shown for the years 2018 (**top** panels) and 2019 (**bottom** panels). On the bottom panels, each arrow indicates the change between the two years: the arrow tail [head] is located on the point $\left(K,K^{*}\right)$ for 2018 [2019]. The countries are represented by their corresponding national flag.

A more synthetic view of the effects of COVID-19 on the rankings can be observed on the PageRank–CheiRank and ImportRank–ExportRank planes displayed in Figure 2. Globally, the countries are located in the vicinity of the diagonal $K=K^{*}$ or $\hat{K}={\hat{K}}^{*}$. Indeed, each country tries to keep, at best, a balance between its import and export volumes. Countries located below the diagonal—i.e., those with $K^{*}<K$ or ${\hat{K}}^{*}<\hat{K}$—are better exporters than importers. Conversely, countries above the diagonal—i.e., those with $K^{*}>K$ or ${\hat{K}}^{*}>\hat{K}$—have better import than export capabilities. Besides providing a summary of the previously discussed results obtained from the top 20 countries displayed in Figure 1, the $\left(K,K^{*}\right)$ or the $\left(\hat{K},{\hat{K}}^{*}\right)$-plane directly allow us to observe the effects of COVID-19 on a broader range; here, with $K,K^{*},\hat{K},{\hat{K}}^{*}\in \left\{1,\ldots ,50\right\}$. From Figure 2, the changes in rankings that are observed from 2018 to 2020 are highlighted by arrows; the tails (tips) of which show the position of countries in 2018 (2020). Figure 2, bottom right panel, shows that the import and export volumes do not vary a great deal during the COVID-19 crisis, with the exceptions of a few cases, such as Russia and Saudi Arabia, which strongly worsened their relative import–export trade balance, or Vietnam and Ireland, which improved it. The changes are larger in the PageRank–CheiRank plane (Figure 2, bottom left panel), highlighting a large-scale rewiring of the WTN as a response to the COVID-19. In addition, Figure A1 in Appendix A clearly shows that, during the pre-COVID-19 period, here from 2018 to 2019, by contrast with the period 2018–2020 (Figure 2), there are no significant variations of country positions on the PageRank–CheiRank plane and in the ImportRank–ExportRank plane.

**Figure 3:**
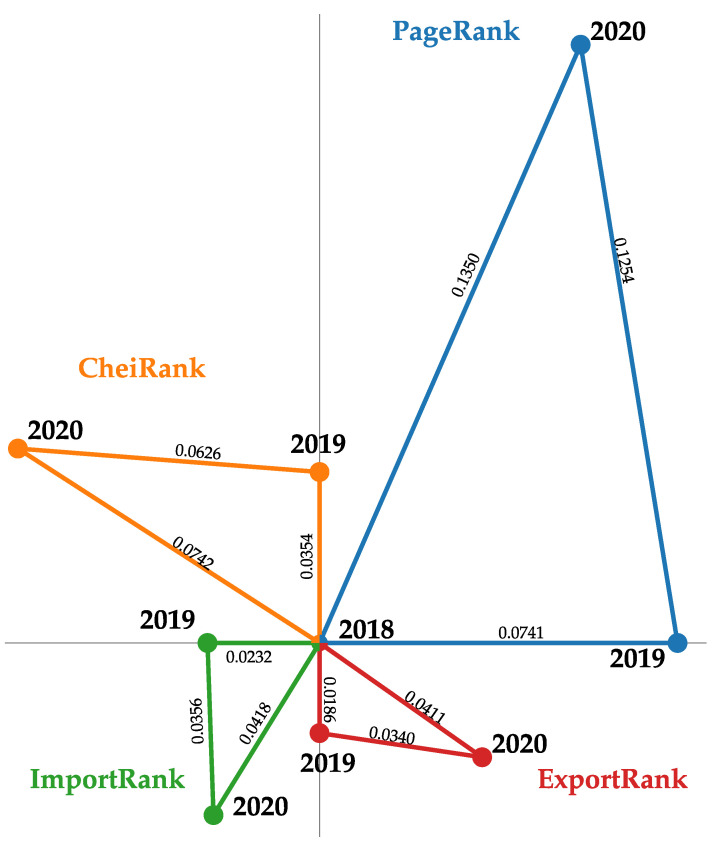
Kendall τ distance between 2018, 2019 and 2020 ranking lists of the 194 world countries. The blue, orange, green and red points correspond to the PageRank, CheiRank, ImportRank and ExportRank country lists, respectively. The Kendall τ distances between ranking lists are mentioned along the segments. For the four rankings, the points corresponding to the 2018 lists are superimposed at the center of the picture.

The Kendall τ distances (see definition in Appendix B) between the ranking lists for the different considered years are shown in Figure 3. We clearly see the influence of COVID-19, since for all the rankings, the 2018–2020 distance and the 2019–2020 distance are on average similar but they are two times greater than the pre-COVID-19 2018–2019 distance. For import (export) metrics, we observe that the distances computed between PageRank (CheiRank) lists are, on average, 3.3 (1.8) greater than those computed between ImportRank (ExportRank) lists. This observation supports again the fact that the influence of COVID-19 is better probed by the Google matrix-based rankings. These results are corroborated by the fact that the number of links decreases by about 20% from 2018 to 2020, whereas the total volume exchanged decreases by about 14%. From 2018 to 2019, these two quantities evolved similarly and dropped by about 3%. In addition, we note that although the ExportRank and the ImportRank triangles have similar sizes, the PageRank triangle is about two times greater than the CheiRank triangle. This suggests that the rewiring induced by the COVID-19 affects in a stronger manner the set of importing countries than exporting countries.

### 3.2. Trade Balances and the COVID-19 Influence

**Figure 4:**
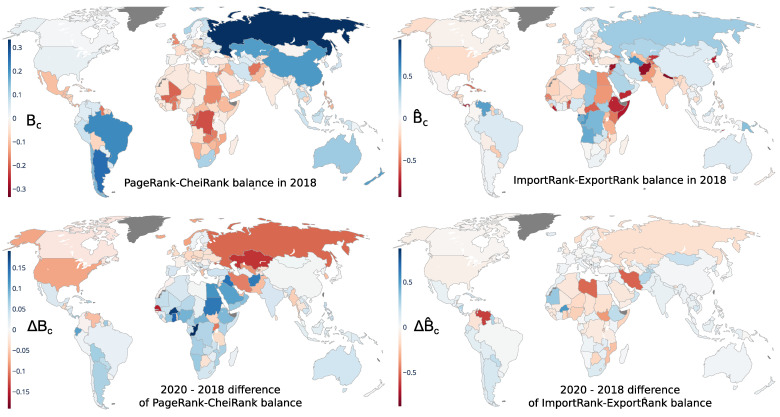
Geographical distributions of the PageRank–CheiRank trade balance $B_{c}$ (**left** panels) and of the ImportRank–ExportRank trade balance ${\hat{B}}_{c}$ (**right** panels). The trade balances for the year 2018 are presented on the first row. The second row displays the difference of trade balance between the years 2018 and 2020; i.e., $\Delta B_{c}=B_{c}^{\left(2020\right)}-B_{c}^{\left(2018\right)}$ (**bottom left** panel) and $\Delta {\hat{B}}_{c}={\hat{B}}_{c}^{\left(2020\right)}-{\hat{B}}_{c}^{\left(2018\right)}$ (**bottom right** panels).

The geographical distributions of the PageRank–CheiRank trade balance $B_{c}$ and of the ImportRank–ExportRank trade balance ${\hat{B}}_{c}$ are shown in Figure 4 for the year 2018. The variations from 2018 to 2020 are also displayed. In 2018, the strongest positive PageRank–CheiRank trade balances $B_{c}$ were obtained (in descending order) for Russia ($B_{\mathrm{RU}}≃0.33$), Argentina, Brazil, China, Kazakhstan, New Zealand, Chile, Australia, Fiji, South Africa and Norway ($B_{\mathrm{NO}}≃0.1$). These countries, several of which belong to the BRICS group, are more efficient exporters than importers of commodities and consequently have a more robust international trade economy than countries with a smaller or even negative PageRank–CheiRank trade balance. The strongest negative PageRank–CheiRank trade balances were obtained for the Democratic Republic of the Congo ($B_{\mathrm{CD}}≃-0.22$), the Republic of the Congo ($B_{\mathrm{CG}}≃-0.2$) and Mali ($B_{\mathrm{ML}}≃-0.19$). In contrast to the ImportRank–ExportRank trade balance geographical distribution (Figure 4, top right panel), which appears inhomogeneous, with strong variations of balance between neighboring countries, the PageRank–CheiRank trade balance geographical distribution (Figure 4, top left panel) appears homogenous at the scale of a continent. For the latter distribution, we observe that negative balances $B_{c}<0$ extend over the African continent (except South Africa), over the region comprising Mexico, the Caribbean and Central America, from the Middle East (except Iran) to South Asia and Central Europe (except Germany). We note also that the PageRank–CheiRank trade balance of the UK is negative, $B_{\mathrm{UK}}≃-0.16$. The positive balance $B_{c}>0$ regions are mainly the Russia–Kazakhstan–China region, Oceania, South America (except Bolivia and the Guyanas) and with lesser importance North America and the north of Europe. A partial explanation of the homogeneity of the positive and negative PageRank–CheiRank trade balance regions could be that an economically virtuous (non-virtuous) country has in general developed trade relations with its neighbors, which take advantage (disadvantage) of the good (bad) economic health of the former. This is a partial explanation in the current globalized world since long distance trade exchanges are, at the least, as important as regional trade exchanges. 

The changes $\Delta B_{c}$ and $\Delta \hat{B_{c}}$ of balances from 2018 to 2020 are shown in the bottom panels of Figure 4 (left and right). We clearly see from the PageRank–CheiRank trade balance variations (Figure 4, bottom left) that countries whose economic health is the most affected by the COVID-19 are, in general, developed countries [45] (with the major exceptions of Australia, New Zealand, Japan, Portugal, Spain, Italy, the Baltic states, Sweden and Finland) and many economies strongly dependent on oil and gas exports, such as those of Russia, Iran, UAE, Venezuela and Trinidad and Tobago, the latter of which has the most affected balance. By contrast, many developing and underdeveloped (mostly African, South Asian, and Sud American countries) increase their PageRank–CheiRank trade balance $B_{c}$. This result is due to the slow down of the global economy driven by the developed countries. Indeed, the variety of the import trades from developed countries towards developing and underdeveloped countries significantly decreased from 2018 to 2020 and automatically enhanced their PageRank–CheiRank trade balance. The most negative ImportRank–ExportRank trade balance variation $\Delta {\hat{B}}_{c}$ is found (in ascending order) for Venezuela (${\hat{B}}_{\mathrm{VE}}≃-0.6$), Iran, Libya and South Sudan (${\hat{B}}_{\mathrm{SS}}≃-0.38$), crudely indicating the bad economic health of these countries.

We argue that the PageRank–CheiRank approach provides a more adequate description of trade balance taking into account the multiplicity of the trade links in contrast to the global volume exchanged description given by the import–export standard approach conventionally used in international trade.

### 3.3. Trade Balance Sensitivity to Product Prices

**Figure 5:**
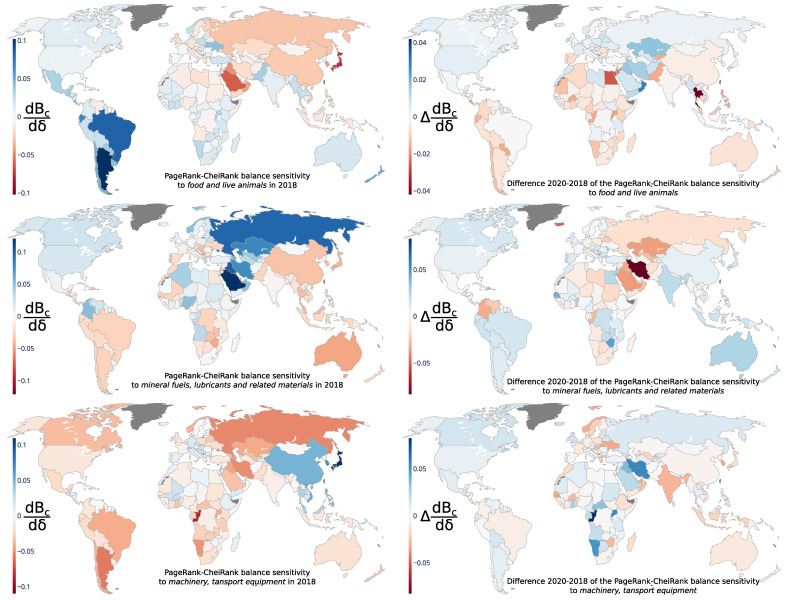
Geographical distributions of the PageRank–CheiRank trade balance sensitivity $dB_{c}/d\delta$ to *food and live animals* (p=0, **top** row), *mineral fuels, lubricants and related materials* (p=3, **middle** row), and *machinery, transport equipment* (p=7, **bottom** row) products. The left column presents the PageRank-CheiRank trade balance sensitivities for the year 2018. The right column presents the difference between the years 2020 and 2018, i.e., $\Delta dB_{c}/d\delta ={\left(dB_{c}/d\delta \right)}^{\left(2020\right)}-{\left(dB_{c}/d\delta \right)}^{\left(2018\right)}$.

The left column of Figure 5 shows the sensitivity $dB_{c}/d\delta$ of the country trade balance $B_{c}$ to a price increase of a group of products p=0, 3, and 7 from Table 1; the right column of Figure 5 gives the variation of sensitivity $\Delta dB_{c}/d\delta ={\left(dB_{c}/d\delta \right)}^{\left(2020\right)}-{\left(dB_{c}/d\delta \right)}^{\left(2018\right)}$ between the years 2018 and 2020.

For *food and live animals* (p=0 in Table 1), the largest positive balance sensitivities $dB_{c}/d\delta$ are found for South and Central America, especially (in ascending order, for Argentina with $dB_{\mathrm{AR}}/d\delta ≃0.1$, Brazil, Ecuador, Chile, and Uruguay with $dB_{\mathrm{UY}}/d\delta ≃0.04$), as well as New Zealand ($dB_{\mathrm{NZ}}/d\delta ≃0.05$) and Ukraine ($dB_{\mathrm{UA}}/d\delta ≃0.04$), whereas the largest negative balance sensitivities $dB_{c}/d\delta$ are found for Japan ($dB_{\mathrm{JP}}/d\delta ≃-0.08$), Arabian Peninsula countries (such as Saudi Arabia with $dB_{\mathrm{SA}}/d\delta ≃-0.06$, Kuwait, Iraq and Oman with $dB_{\mathrm{OM}}/d\delta ≃-0.04$), and Russia and China with $dB_{\mathrm{RU}}/d\delta ≃dB_{\mathrm{CN}}/d\delta ≃-0.03$. The former (latter) set of countries would have benefited from (been disadvantaged by) a price or a volume exchange increase of *food and live animals*. We attribute the variation of the sensitivity $\Delta dB_{c}/d\delta$ to COVID-19, which induced a positive increase of the balance trade sensitivity for Oman, Iraq, Iran and Kazakhstan and a negative decrease for Thailand, Egypt and Jordan.

For *mineral fuels, lubricants and related materials* (p=3 in Table 1), the largest positive balance sensitivities are found (in descending order) for Saudi Arabia ($dB_{\mathrm{SA}}/d\delta ≃0.12$), Iraq, Russia, Kuwait, Qatar, Kazakhstan, Iran, Azerbaijan and the UAE ($dB_{\mathrm{AE}}/d\delta ≃0.53$) which are world-leading petroleum and gas producers. In contrast, the negative sensitivities are at the least equal to −0.047 for Australia and are distributed mostly over all countries which are not major oil and petroleum exporters. The largest positive variations $\Delta dB_{c}/d\delta$ are obtained, among the large countries, for Australia, India and Egypt, which are attributed to a reduction of petroleum imports due to COVID-19’s reduction of production. The largest negative variation $\Delta dB_{c}/d\delta$ is for Iran, Kazakhstan and Saudi Arabia, which suffered from a reduction in petroleum demand.

For *machinery and transport equipment* (p=7 in Table 1), the largest positive balance sensitivities are obtained (in descending order) for Japan ($dB_{\mathrm{JP}}/d\delta ≃0.11$), South Korea, Vietnam and China ($dB_{\mathrm{CN}}/d\delta ≃0.052$); the largest negative values are obtained (in ascending order) for Congo ($dB_{\mathrm{CG}}/d\delta ≃-0.085$), Argentina, Cyprus, Russia and Iran ($dB_{\mathrm{IR}}/d\delta ≃-0.051$). We also observe a positive relative sensitivity to *machinery and transport equipment* for European countries. Many of these countries in addition to India and South Korea have a negative variation of sensitivity due to COVID-19.

**Figure A2:**
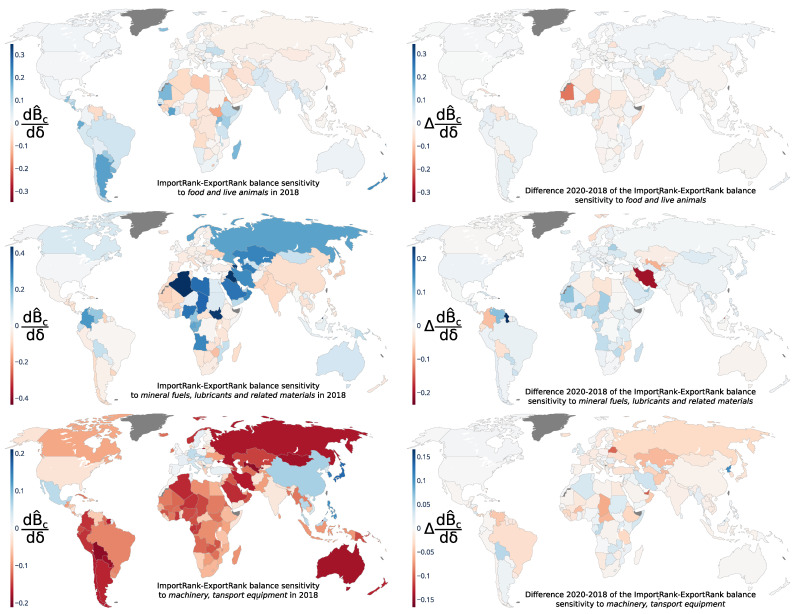
Geographical distributions of the ImportRank–ExportRank trade balance sensitivity $d{\hat{B}}_{c}/d\delta$ to *food and live animals* (**top** row), *mineral fuels, lubricants and related materials* (**middle** row), and *machinery and transport equipment* (**bottom** row) products. The left column presents the PageRank–CheiRank trade balance sensitivities for the year 2018. The right column presents the difference between the years 2020 and 2018; i.e., $\Delta d{\hat{B}}_{c}/d\delta ={\left(d{\hat{B}}_{c}/d\delta \right)}^{\left(2020\right)}-{\left(d{\hat{B}}_{c}/d\delta \right)}^{\left(2018\right)}$.

For the convenience of readers, the geographical distribution of the trade balance sensitivity obtained from the ImportRank and ExportRank is also given in Appendix A (see Figure A2). We do not discuss here this figure, since as explained above this approach is less informative.

### 3.4. PageRank–CheiRank Product Trade Balance

**Figure 6:**
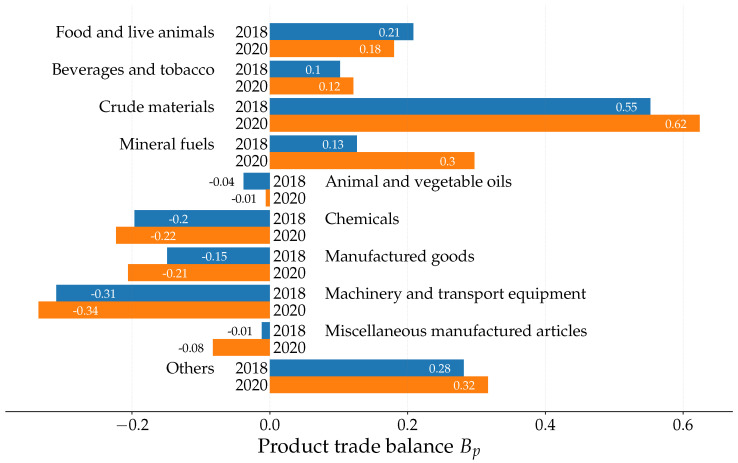
PageRank-CheiRank product trade balance $B_{p}$ for years 2018 (blue bars) and 2020 (orange bars); the product balance ${\hat{B}}_{p}$ computed from ImportRank-ExportRank is zero.

It is interesting to analyze the trade balance not only for countries but also for products performing a summation of $B_{pc}$ over countries. This peculiarity is not possible in the case of the ImportRank–ExportRank approach, since, by definition, the ImportRank–ExportRank product trade balance is zero, ${\hat{B}}_{p}=\sum_{c}{\hat{B}}_{pc}=0$. Indeed, after summation over countries, the export and import probabilities—i.e., the relative export and import volumes—are equal, ${{\hat{P}}^{*}}_{p}={\hat{P}}_{p}$. However, within the PageRank–CheiRank approach, the probabilities $P_{p}^{*}$ and $P_{p}$ are different, and thus the product trade balance of some products, $B_{p}=\sum_{c}B_{pc}$, can be positive and negative for some others. The trade balances for each group of products (Table 1) are presented in Figure 6 for the years 2018 and 2020. The balance $B_{p}$ is positive for products p=0,1,2,3,9, and hence they are more export oriented, $P_{p}^{*}>P_{p}$. These products are more central in the inverted WTN than in the direct WTN. Otherwise stated, the export abilities of these products exceed their import abilities. In contrast, the products p=4,5,6,7,8 have negative balances and are import oriented, $P_{p}>P_{p}^{*}$. These products’ import abilities supersede their export abilities. The PageRank–CheiRank approach allows us to infer a kind of structural law of supply and demand. The strongest variation of product balance from 2018 to 2020 takes place for *mineral fuels* (p=3), with an increase of $B_{p}$ by a factor 2.3 from 2018 to 2020, while the strongest negative decrease of $B_{p}$ is observed for *manufactured goods* (p=6) by a factor of 1.4 from 2018 to 2020. An even stronger negative decrease of $B_{p}$ takes place for *miscellaneous manufactured articles* (p=8) with a factor of 8; however, for this product, the balance $B_{p}$ is relatively small. The effects of COVID-19 are salient here. The products *manufactured goods* and *miscellaneous manufactured articles* lost their export ability during 2020, whereas import abilities were maintained. Conversely, export abilities were maintained for *mineral fuels*, but structural demand declined in 2020.

**Figure 7:**
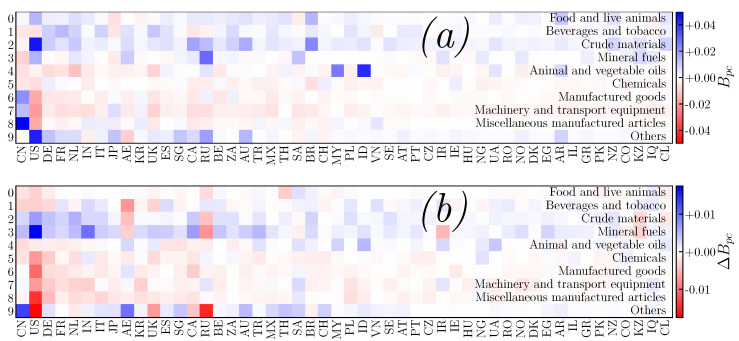
PageRank-CheiRank (product, country) trade balance $B_{pc}$ (panel (**a**)) for the year 2018. Trade balance differences between years 2020 and 2018 $\Delta B_{pc}=B_{pc}^{\left(2020\right)}-B_{pc}^{\left(2018\right)}$ are presented in panel (**b**). The columns correspond to countries *c*, sorted according to 2DRank (see Table 2 for the first 20), and rows correspond to products *p* (see Table 1). The meaning of the ISO 3166-1 alpha-2 country codes not present in Table 2 can be found, e.g., at [46].

**Figure A3:**
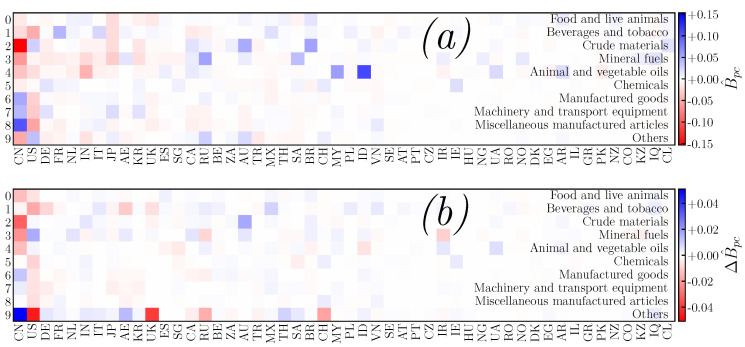
ImportRank-ExportRank (product, country) trade balance ${\hat{B}}_{pc}$ (panel (**a**)) for the year 2018. Trade balance differences between years 2020 and 2018—i.e., $\Delta {\hat{B}}_{pc}={\hat{B}}_{pc}^{\left(2020\right)}-{\hat{B}}_{pc}^{\left(2018\right)}$—are presented in panel (**b**). The columns correspond to countries *c*, sorted according to 2DRank (see Table 2 for the first 20), and rows correspond to products *p* (see Table 1). The meaning of the ISO 3166-1 alpha-2 country codes not present in Table 2 can be found, e.g., at [46].

The distribution of product trade balance by countries, $B_{pc}$, obtained from the PageRank–CheiRank approach, is shown in Figure 7 for 2018. The variations, $\Delta B_{pc}$, from 2018 to 2020 of the product trade balances per country are also shown (the results obtained from the ImportRank–ExportRank approach are given in the Appendix A, see Figure A3). In 2018, China has a positive trade balance $B_{pc}$ for products p=6, 7 and 8, with the most negative trade balance $B_{pc}$ is found for product p=3. The product trade balance variation from 2018 to 2020 is significantly positive for product p=9 (which includes financial transactions) and slightly positive (negative) for p=3 (p=2 and 1). The COVID-19-related variation of the USA product trade balance from 2018 to 2020 is significantly negative for products p=4, 5, 7, 8 and 9 and positive for products p=2 and 3. Among significant positive values of $B_{pc}$ for the other countries in Figure 7a, we highlight Indonesia and Malaysia for p=4, Russia for p=3 and Brazil for p=2; the negative product trade balances are less pronounced. Among the most significant variations $\Delta B_{pc}$ in Figure 7b, for other countries, we highlight India, with a positive value for p=3, the United Arab Emirates with positive p=9 and negative p=1, and a negative variation for Russia with p=9, 3 and 2. For interested readers, the same figure as Figure 7 but obtained from ImportRank–ExportRank can be found in Appendix A (see Figure A3).

### 3.5. COVID-19 Induced Rewiring of the WTN

**Figure A4:**
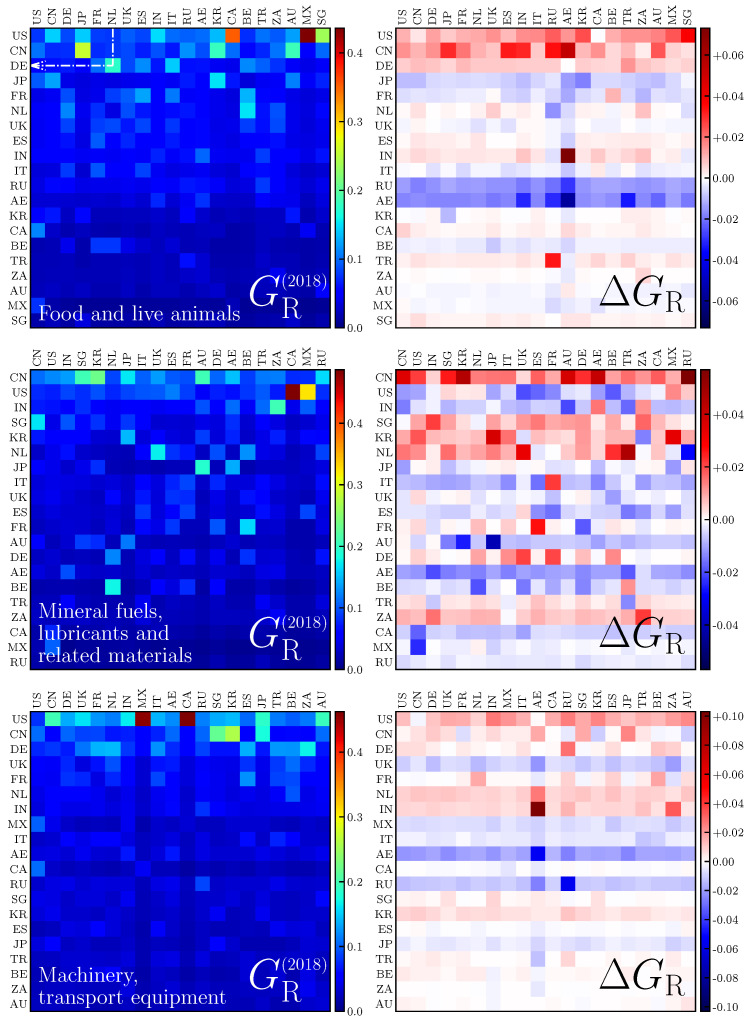
Reduced Google matrices associated with *food and live animals* (**top** row), *mineral fuels, lubricants and related materials* (**middle** row) and *machinery and transport equipment* (**bottom** row) products and for the 20 countries at the top of the PageRank and CheiRank lists for the multiproduct WTN (see Table 2). The left column presents the reduced Google matrices $G_{R}^{\left(2018\right)}$ concerning the trade exchanges of the year 2018, and the right column presents the difference of reduced Google matrices between the years 2020 and 2018; i.e., $\Delta G_{R}=G_{R}^{\left(2020\right)}-G_{R}^{\left(2018\right)}$. The matrix entries correspond to $\left(c,p\right)$ pairs; i.e., $\left(c,p=0\right)$ for the top row, $\left(c,p=3\right)$ for the middle row, and $\left(c,p=7\right)$ for the bottom row. These $\left(c,p\right)$-pairs are sorted according to the PageRank algorithm (the country ranks are different for each product *p*). The broken arrow on the top left panel shows how to read such a matrix. The arrow emanates from the country NL column-index, goes down and turn left towards the country DE row-index. The color at the turning point gives the value, here ∼0.2, of the ${G_{R}}_{\left(\mathrm{DE}\;p=0,\mathrm{NL}\;p=0\right)}$ matrix element. This matrix element is a measure of the ability of Germany to import *food and live animals* from the Netherlands. In comparison with the other elements of the NL column, this ability is the greatest among the other 19 considered countries. Hence, the most efficient importer of *food and live animals* from the Netherlands is Germany.

**Figure A5:**
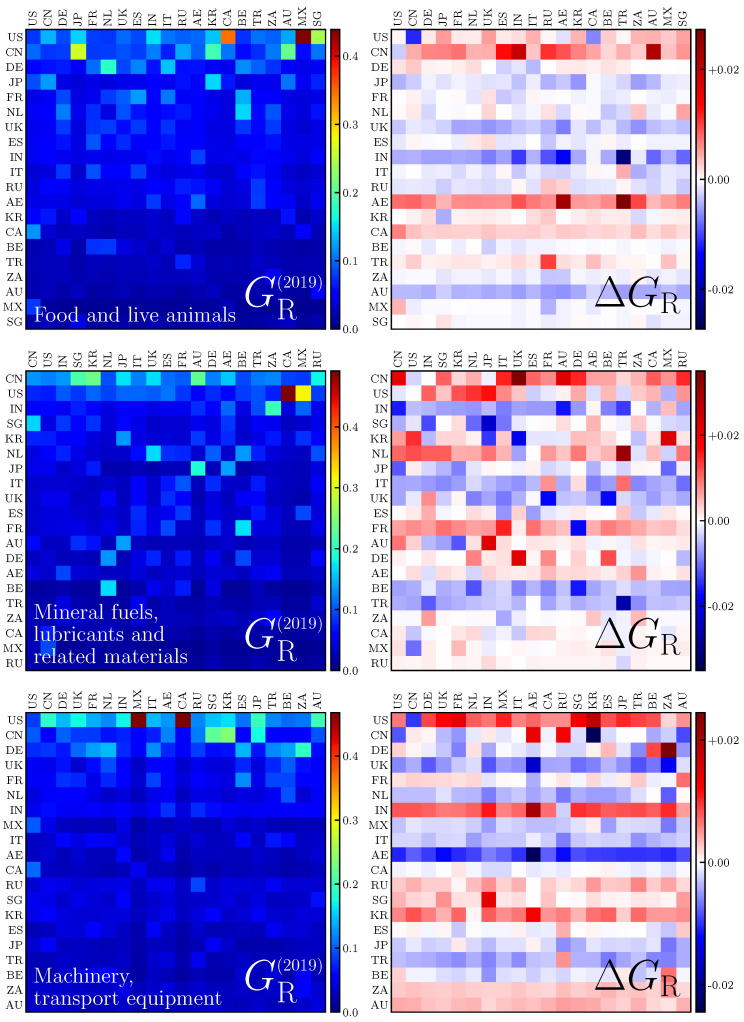
Same as Figure A4 but for the years 2018 and 2019.

In Figure A4, we show the three reduced Google matrices $G_{R}$ for the subset of 20 countries with top PageRank and CheiRank indexes (see Table 2) for three selected products p=0,3,7 in the year 2018. Such a matrix $G_{R}$ describes the effective trade flows of a given product between these 20 countries. We also present the variation $\Delta G_{R}=G_{R}^{\left(2020\right)}-G_{R}^{\left(2018\right)}$ of the matrix elements between 2018 and 2020, which we attribute to the impact of COVID-19. Indeed, the variations over a year in the pre-COVID-19 period are two to five times less significant than the variations observed between 2018 and 2020 (see Figure A5 in Appendix A). The above described REGOMAX algorithm takes into account all the indirect chains of trade transactions between these 20 countries in the global WTN (1940 nodes, 449675 trade transactions over the considered period).

From the reduced Google matrix $G_{R}$, we easily see the strongest flows of a selected product between the countries in 2018. Thus, for *food and live animals*, the strongest trade transactions are from Mexico to the USA, Canada to the USA, Japan to China and Netherlands to Germany. For *mineral fuels*, the strongest ones are Canada to the USA and Mexico to the USA, and the same is found for *machinery*. Here, we see the domination of the USA, which has the top PageRank index K=1 in 2018 and hence the strongest import flows from certain countries (especially from its neighbors Mexico and Canada).

The matrix elements of the variation $\Delta G_{R}$ from 2018 to 2020 have a richer structure. Thus, for *food and live animals*, the strongest positive variations are observed from the UAE to India and to China; among the negative ones, we highlight variations from Turkey, Russia and India to the UAE. For *mineral fuels*, the strongest positive variations are obtained from Russia and South Korea to China, from Turkey to the Netherlands; among the negative ones, we highlight trade transactions from Japan and South Korea to Australia and from Russia to the Netherlands. Thus, we see that from 2018 to 2020, Russia is changing its *mineral fuels* trade flows by increasing its flow towards China and decreasing it towards the EU; represented, here, by the Netherlands. For *machinery* products, we highlight among the strongest variations a positive one from the UAE to India and a negative one from South Africa to the UK.

**Figure 8:**
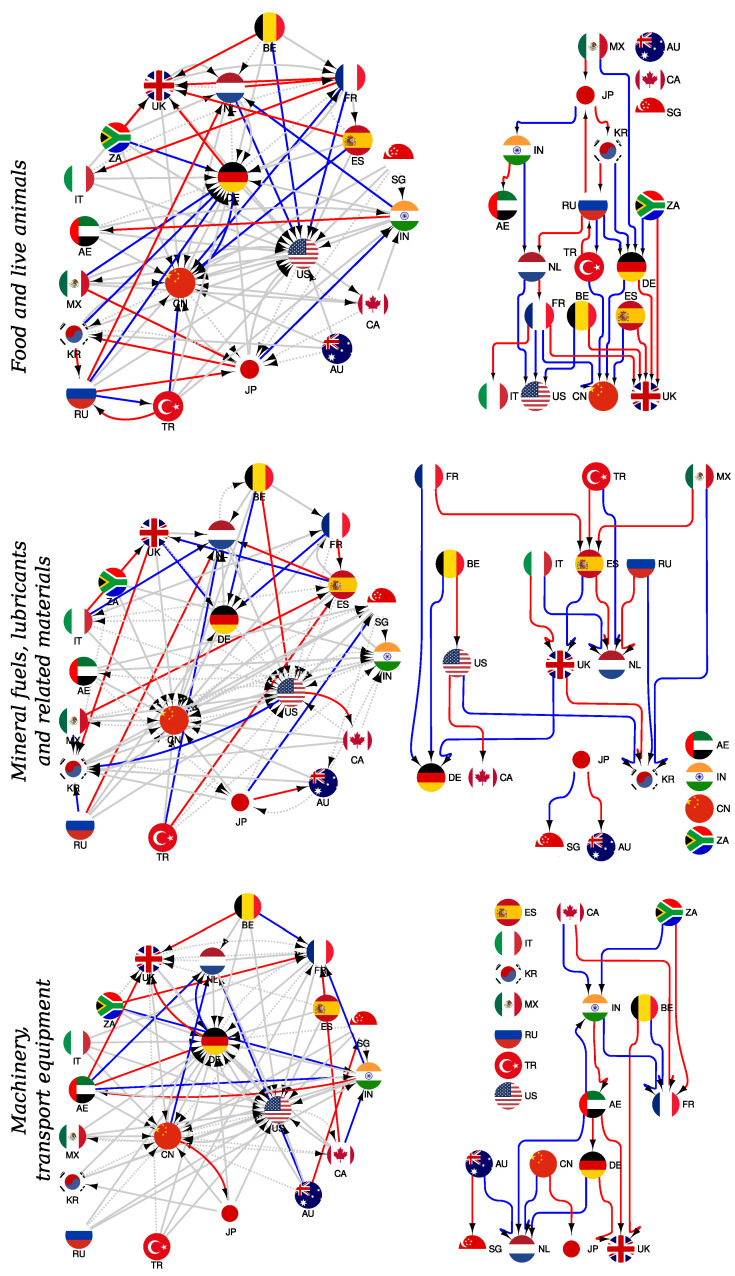
Reduced networks for *food and live animals* (**top**), *mineral fuels, lubricants and related materials* (**middle**) and *machinery and transport equipment* (**bottom**) trade exchanges. In the left panel, the reduced networks of the top 20 most efficient exporters or importers according to the PageRank and CheiRank algorithms are presented (see Table 2). For each panel, data from the years 2018 and 2020 are combined. For each year and from each country, the four most important export transitions encoded in the $G_{R}$ are kept. The gray arrows correspond to important export transitions existing in both years 2018 and 2020. The blue arrows correspond to important export transitions appearing in 2020 but absent in 2018. Conversely, the red arrows correspond to important export transitions existing in 2018 but disappearing in 2020. The solid (dashed) gray arrows are associated with an increase (a decrease) of the corresponding $G_{R}$ matrix element from 2018 to 2020. On the right panels, only the differences between 2020 and 2018 are highlighted (i.e., only blue and red arrows are kept). Countries are represented by their national flag.

In summary, the REGOMAX approach allows us to find the most significant trade flows between a selected group of countries for a specific product. The matrix presentation of such trade flows can be represented by a reduced network (Figure 8). Let us consider the reduced Google matrix associated with *food and live animals* (Figure A4, top panel). For each column *c*, we select the four largest values corresponding to the four strongest export trade flows from country *c* to other countries $c^{\prime }$. This allows us to select the most important commercial partners $c^{\prime }$ importing *food and live animals* products from the country *c*. Hence, from each country of the considered set of interest—i.e., here, the 20 most important actors of international trade—we are able to draw four directed links towards four other countries. The obtained reduced network highlights the principal *food and live animals* trade interactions between the considered countries. The advantage of the reduced network resides in the fact that, besides direct trade interactions, it displays also long-range trade interactions between the commercial partners. The three panels of Figure 8 present the reduced networks for *food and live animals*, for *mineral fuels, lubricants and related materials*, and for *machinery and transport equipment* products, respectively. More precisely, for each type of products, in Figure 8 left panels, the reduced networks for the years 2018 and 2020 are jointly displayed: red (blue) links highlight trade interactions disappearing (arising) in 2020 and (not) present in 2018, while the solid (dashed) gray links are stable trade interactions whose weights increase (decrease) from 2018 to 2020. For *food and live animals*, we observe that the main importers are the USA, China, Germany, Japan, France and the UK. From the top right panel of Figure 8, only the changes from 2018 to 2020 are displayed, allowing us to directly observe the WTN rewiring induced by COVID-19. From 2018 to 2020, we clearly observe an increase of trade flows towards the USA, China and Germany, but a severe decrease towards the UK. The latter loses importing flows which were redirected, in 2020, mainly to China and to Germany. For *mineral fuels, lubricants and related materials*, the trade flows are polarized towards the USA, China, India, Singapore, South Korea, Germany and the Netherlands. From the middle right panel of Figure 8, we observe an increase of trade flows towards Germany and South Korea, whereas Spain loses trade flows to the benefit of South Korea, the Netherlands and Germany. For *machinery and transport equipment*, importing flows are directed towards the USA, Germany, China, France, India and the UK. From the bottom right panel, we observe an increase of the importance of the Netherlands at the expense of Singapore, Japan and the UK. In contrast, the UK loses importance to the benefit of the Netherlands and France.

## 4. Discussion

In summary, on the basis of the UN Comtrade database gathering data of international trade [13], we constructed the multiproduct WTN for the years 2018 to 2020. We presented its Google matrix analysis based on the PageRank algorithm [14,15] and other extended algorithms described in [25,26,28,30,31]. The PageRank–CheiRank approach allows us to obtain a detailed and advanced description of international trade, in comparison to the usual import–export analysis, since it takes into account the complex multiplicity of the trade transactions between the countries. Thus, the PageRank–CheiRank method properly takes the robustness and the diversity of the trade connections of a given pair (country, product) into account, highlighting the network influence of specific countries, which is not so visible from the standard import and export description. As an example, the rankings of the countries with respect to the gas and oil trade are rather different depending on the chosen analysis, either the PageRank–CheiRank analysis or the usual ImportRank–ExportRank analysis. We highlight that, in 2018, Russia is at the $K^{*}=4$ CheiRank position in contrast to the ${\hat{K}}^{*}=9$ ExportRank position. As the CheiRank takes account of the structure of the WTN, this significant difference is attributed to the diversity of the trade relations of Russia directed to both Asian and European countries.

Using the PageRank–CheiRank algorithms, we determine significant ranking changes of world countries from 2018 to 2020 which are attributed to the impact of COVID-19 on the WTN (since there are only small changes from 2018 to 2019 in absence of the pandemic). This approach also determines the trade balance $B_{c}$ of all world countries in 2018 and 2020, in a manner more adequate than the crude import–export treatment. The balance variation $\Delta B_{c}$ between 2018 and 2020 shows that the most negatively affected large countries are Kazakhstan, Russia, Iran and the USA, which have been impacted by the global reduction of world production and the related import of *mineral fuels*. The PageRank–CheiRank description also allows us to determine the sensitivity of the trade balance to the price or volume increase of a specific type of product and its variation due to COVID-19.

New features are also discovered for the trade balance $B_{p}$ of a product *p*. In contrast to the import–export description which, by definition, gives 0 for the balance of each product, our analysis shows that some products are export-oriented—*food and live animals* (p=0), *beverages and tobacco* (p=1), *crude materials* (p=2), *mineral fuels* (p=3) and *other transactions* (p=9)— while others are import-oriented—*animal and vegetable oils* (p=4), *chemicals* (p=5), *manufactured goods* (p=6), *machinery and transport equipment* (p=7) and *miscellaneous manufactured articles* (p=8). We also determine the variations of these product balances $B_{p}$ induced by COVID-19. Thus, due to COVID-19, the product balance is significantly increased for p=2 and 3 products and significantly decreased for p=6, 7 and 8 products. We attribute this to a significant import reduction of products p=2 and 3, since due to COVID-19, the production and hence exports of p=6, 7 and 9 products were reduced.

Finally, the REGOMAX method, which allows us to determine the reduced Google matrix $G_{R}$ for selected countries and products, determines the most COVID-affected trade flows and provides a clear graphical network structure, highlighting the rewiring of the WTN induced by the COVID-19 pandemic.

Our results, obtained from the UN Comtrade database, demonstrate a strong impact of COVID-19 on international trade. We argue that there are multiple social and economic origins of this negative impact: the pandemic significantly reduced the travel of people and goods between countries, reducing requests for petroleum and gas. In Figure 7b, the trade balance in *mineral fuels* dropped for the UAE, Russia, Saudi Arabia and Iran, and only the USA and India (among the largest countries) improved their balance. The production of specific products was reduced due to illness of people, and hence the balance for *machinery* and *manufactured articles* induced a negative product balance for USA and almost all the European countries; only China was not perturbed (almost no balance changes) in the context of these groups of products. Indeed, most of these products were produced in Asia and were absent in Europe (e.g., not enough masks in Europe and not enough medicines). The impact of COVID-19 clearly showed that the industrial production in USA and EU countries should be increased in order to become more self-sufficient and independent—a fact that should be taken into consideration by policy makers. Our studies are based on the years 2018–2020, and it will be useful to perform the analysis for a larger number of years. Furthermore, as stated in [27], the UN Comtrade database does not provide information about a transfer of one product to another (e.g., one needs steel to produce cars and other road machines), which is an important element of the economy’s cycle. Thus, it would be very useful to add such information to the UN Comtrade database or to use a detailed value chain network, which is still lacking at the global scale.

## Figures and Tables

**Table 1:** List of product groups from the Standard International Trade Classification (SITC) [13].

SITC Code	Standard International Trade Classification (SITC) Product Groups
0	Food and live animals
1	Beverages and tobacco
2	Crude materials, inedible, except fuels
3	Mineral fuels, lubricants and related materials
4	Animal and vegetable oils, fats and waxes
5	Chemicals and related products, n.e.s.
6	Manufactured goods classified chiefly by material
7	Machinery and transport equipment
8	Miscellaneous manufactured articles
9	Commodities and transactions not classified elsewhere in the SITC

**Table 2:** List of top 20 most efficient exporting or importing countries according to the PageRank and CheiRank algorithms. These 20 countries are located in the $\left[1,22\right]\times \left[1,22\right]$ square of the $\left(K,K^{*}\right)$-plane (see Figure 2). These 20 countries are sorted using the 2DRank indexes $K_{2}$ [17]; i.e., by ascending value of $\max\left(K,K^{*}\right)$, and then, in case of *ex-æquo*, by ascending value of $K^{*}$. The column CC gives the ISO 3166-1 alpha-2 country codes (see e.g., [46]).

$K_{2}$	*K*	$K^{*}$	CC	Country
1	2	1	CN	China
2	1	2	US	United States
3	3	3	DE	Germany
4	4	6	FR	France
5	7	5	NL	Netherlands
6	6	7	IN	India
7	8	9	IT	Italy
8	10	8	JP	Japan
9	9	10	AE	United Arab Emirates
10	12	11	KR	South Korea
11	5	12	UK	United Kingdom
12	11	15	ES	Spain
13	16	13	SG	Singapore
14	13	16	CA	Canada
15	17	4	RU	Russia
16	14	19	BE	Belgium
17	20	18	ZA	South Africa
18	21	17	AU	Australia
19	18	21	TR	Turkey
20	15	22	MX	Mexico

## Data Availability

The world trade data are available at https://comtrade.un.org (accessed on 2 December 2021).

## Abbreviations

The following abbreviations are used in this manuscript:

COVID-19	Coronavirus disease 2019
WTN	World Trade Network
WTO	World Trade Organization
UN	United Nations
WWW	World Wide Web
SITC	Standard International Trade Classification
REGOMAX	Reduced Google matrix
USD	United States Dollar
USA	United States of America
UAE	United Arab Emirates
BRICS	Brazil, Russia, India, China, South Africa
UK	United Kingdom
CC	Country code
EU	European Union

## Appendix A. Supporting Figures

Here, we present five additional figures supporting the results described in the main part of the paper.

## Appendix B. Kendall τ Distance

Let us assume *N* elements labeled by an integer $\left\{1,\ldots ,N\right\}$. Let us assume two ranking lists $\tau_{1}$ and $\tau_{2}$. The element *i* occupies the positions $\tau_{1}\left(i\right)$ and $\tau_{2}\left(i\right)$ in these two lists. The Kendall τ distance between the ranking lists $\tau_{1}$ and $\tau_{2}$ is defined as

(A1) $$d\left(\tau_{1},\tau_{2}\right)={\left(N\left(N-1\right)\right)}^{-1}\sum_{i<j}\left(1-\mathrm{sign}\left(\tau_{1}\left(i\right)-\tau_{1}\left(j\right)\right)\mathrm{sign}\left(\tau_{2}\left(i\right)-\tau_{2}\left(j\right)\right)\right)$$

where the sum is performed over all the different pairs (i,j) of elements and where $\mathrm{sign}\left(x\right)=x/\left|x\right|$. The Kendall τ distance $d\left(\tau_{1},\tau_{2}\right)$ ranges from 0 for identical lists, $\tau_{1}\equiv \tau_{2}$, to 1 if the $\tau_{1}$ list is the reverse of the $\tau_{2}$ list, and vice versa.
